# Sphingolipid Metabolic Pathway: An Overview of Major Roles Played in Human Diseases

**DOI:** 10.1155/2013/178910

**Published:** 2013-08-04

**Authors:** Raghavendra Pralhada Rao, Nanditha Vaidyanathan, Mathiyazhagan Rengasamy, Anup Mammen Oommen, Neeti Somaiya, M. R. Jagannath

**Affiliations:** Biology Group, Connexios Life Sciences Private Limited, No.49 Shilpa Vidya, First Main Road, 3rd Phase JP Nagara, Bangalore 560078, India

## Abstract

Sphingolipids, a family of membrane lipids, are bioactive molecules that participate in diverse functions controlling fundamental cellular processes such as cell division, differentiation, and cell death. Given that most of these cellular processes form the basis for several pathologies, it is not surprising that sphingolipids are key players in several pathological processes. This review discusses the role of the sphingolipid metabolic pathway in diabetes, Alzheimer's disease, and hepatocellular carcinoma, with a special emphasis on the changes in gene expression pattern in these disease conditions. For convenience, the sphingolipid metabolic pathway is divided into hypothetical compartments (modules) with each compartment representing a physiological process and changes in gene expression pattern are mapped to each of these modules. It appears that alterations in the gene expression pattern in these disease conditions are biased to manipulate the system in order to result in a particular disease.

## 1. Introduction

**Figure 1:**
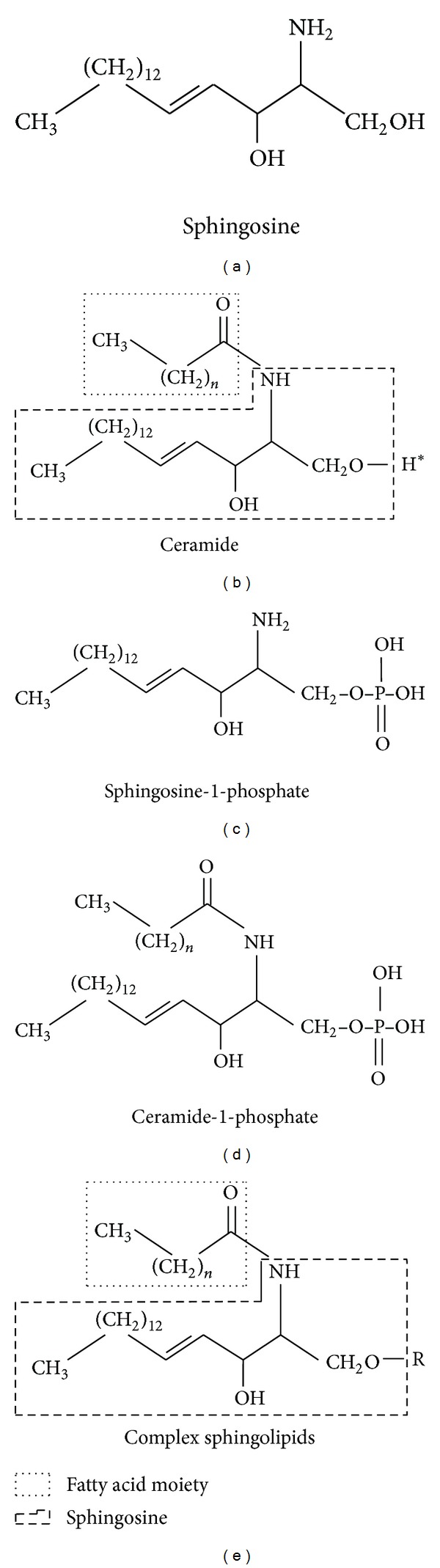
Structure of key sphingolipid molecules. All sphingolipids are comprised of a sphingoid base, and in mammals sphingosine (mainly C-18) is the major sphingoid base (a). A long chain fatty acid attached to sphingosine through amide linkage forms ceramide (b). Complex sphingolipids are obtained by replacement of hydrogen group of ceramide (H*) with various functional head groups (represented as R group in (e)). Complex sphingolipids vary in the nature of the polar head groups. For example, in sphingomyelin the head group is phosphocholine whereas in glycosphingolipids the head group could be one or more sugar residues. The phosphorylated derivatives, namely, sphingosine-1-phosphate (c) and ceramide 1 phosphate (d), are obtained by action of respective kinases on sphingosine and sphingosine-1-phosphate.

Sphingolipids are a class of natural lipids comprised of a sphingoid base backbone, sphingosine. Sphingosine N-acylated with fatty acids forms ceramide [1], a central molecule in the sphingolipid biology. A variety of charged, neutral, phosphorylated, or glycosylated moieties are attached to ceramide further forming complex sphingolipids [2] (see Figure 1 for details). For example, phosphoryl choline attached to ceramide makes the most abundant mammalian sphingolipid, sphingomyelin. These moieties result in both polar and nonpolar regions giving the molecules an amphipathic character which accounts for their tendency to aggregate into membranous structures. Furthermore, such variations found in their chemical structures allow them to play diverse roles in cellular metabolism. Research in the past decade has clearly indicated that sphingolipids are not just the structural components of cell membrane but also act as signaling molecules controlling a majority of cellular events including signal transduction, cell growth, differentiation, and apoptosis [3–5]. Ceramide, sphingosine, sphingosine-1-phosphate (S1P), and ceramide-1-phosphate (C1P) have emerged as chief bioactive mediators in the context of sphingolipid biology. 

Although sphingolipids contribute to only a small proportion of the total cellular lipid pool, their accumulation in certain cells may be a trigger for pathology of many diseases. Because of the presence of a highly integrated metabolic network among various bioactive sphingolipids, it can be implicated that manipulation of one enzyme or metabolite may result in unexpected changes in metabolite levels, enzyme activities, and cellular programs [6, 7]. 

Whilst the scientific literature has been enriched by articles focusing on structural diversity and cellular metabolism, this review focuses on how alterations in expression of genes involved in sphingolipid metabolism could result in the progression of severe diseases. We chose three most prevalent diseases: type 2 diabetes, Alzheimer's disease, and hepatocellular carcinoma and analyzed the nature of gene expression changes in these disease conditions. The gene expression changes were further translated into possible physiological effects and these effects were analyzed to check for any correlation to the key pathology of the disease in question. It turns out that, under these disease conditions at least, the sphingolipid metabolic pathway is modulated in such a way that the resultant changes in the physiology act as a significant contributor to the disease pathology. 

## 2. Sphingolipid Metabolism: An Overview

**Figure 2:**
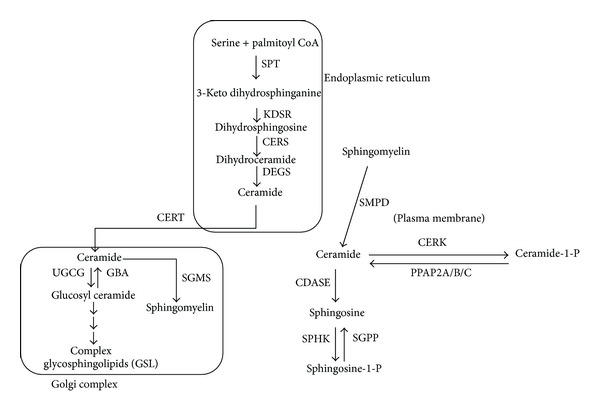
Key reactions involved in the sphingolipid metabolic pathway. Ceramide is produced in the ER and later transported to the Golgi complex for further conversion to complex sphingolipids. In addition to *de novo* synthesis, ceramide is also generated by hydrolysis of sphingomyelin. Ceramide is subject to conversion to various other sphingolipid intermediates like ceramide-1-phosphate, sphingosine, and sphingosine-1-phosphate. Cellular compartments are represented by boxes and enzymes are italicized. CDASE: ceramidase; CERK: ceramide kinase; CERS: ceramide synthase; CERT: ceramide transfer protein; DEGS: dihydroceramide desaturase; ER: endoplasmic reticulum; GBA: glucosyl ceramidase; GC: Golgi complex; KDSR: 3-keto dihydrosphinganine reductase; PM: plasma membrane; PPAP2A/B/C: phosphatidic acid phosphatase 2A/B/C; SGMS: sphingomyelin synthase; SGPP: sphingosine-1-phosphate phosphatase; SMPD: sphingomyelin phosphodiesterase; SPHK: sphingosine kinase; SPT: serine palmitoyll transferase; UGCG: UDP-glucose ceramide glucosyltransferase.

The process of metabolism of sphingolipids has been studied extensively and most of the biochemical pathways of synthesis and degradation, including all the enzymes involved, have been determined successfully [8]. Sphingolipid metabolic pathway is an important cellular pathway that represents a highly coordinated system linking together various pathways, where ceramide occupies a central position in both biosynthesis and catabolism, thereby crafting a metabolic hub [9]. The reaction sequences involved in the formation of ceramide and other sphingolipids are represented in Figure 2. 

### 2.1. **De Novo ** Synthesis

The first step in the *de novo * biosynthesis of sphingolipids is the condensation of serine and palmitoyl CoA, a reaction catalyzed by the rate-limiting enzyme, serine palmitoyltransferase (SPT, EC 2.3.1.50), to produce 3-ketodihydrosphingosine [10, 11]. Among various organisms, several metabolic divergences appear after the formation of sphinganine (dihydrosphingosine). In fungi and higher plants, sphinganine thus formed is first hydroxylated to phytosphingosine and then acylated to produce phytoceramide, whereas in animal cells sphinganine it is acylated to dihydroceramide which is later desaturated to form ceramide [12]. These reactions leading to generation of ceramide starting from serine take place in the endoplasmic reticulum. Ceramide thus generated needs to be transported to the Golgi complex, where it serves as a substrate for production of complex sphingolipids like sphingomyelin and glycosphingolipids (Figure 2). Both vesicular and nonvesicular transport mechanisms can mediate this process. The non-vesicular transport is mediated by the ceramide transfer protein (CERT) in mammals, in an ATP-dependent manner [13]. Once transported to the Golgi complex, several different head groups can be added to ceramide to form different classes of complex sphingolipids [14]. These complex sphingolipids will traverse different cellular locations mainly through vesicular transport.

### 2.2. Ceramide Homeostasis

Ceramide is considered as a molecule central to sphingolipid metabolic pathway and it serves as a branch point in the pathway. It acts as substrate not only for complex sphingolipids but also for the generation of ceramide-1-phosphate (C1P) and sphingosine, and sphingosine can be further converted into sphingosine-1-phosphate (S1P) (reactions are outlined in Figure 2). Various secondary signaling intermediates produced by further conversion of ceramide can participate in diametrically opposite cellular processes; for example, ceramide and sphingosine are proapoptotic while their phosphorylated derivatives, C1P and S1P, are involved in progrowth activities [15]. Several studies have established that ceramide and sphingosine function as tumor-suppressor lipids mediating apoptosis, growth arrest, senescence, and differentiation. On the other hand, S1P and C1P are regarded as a tumor-promoting lipids involved in cell proliferation, migration, transformation, inflammation, and angiogenesis [16–19].

In addition to *de novo* biosynthesis, ceramide can be generated in the cell through hydrolysis of complex sphingolipids. The hydrolytic pathway controls the regeneration of ceramide from the complex sphingolipid pool, for example, from glycosphingolipids (GSLs) and sphingomyelin (SM), through the action of specific hydrolases and phosphodiesterases. Regeneration of ceramide from sphingomyelin is carried out by the plasma membrane bound enzyme SMPD (sphingomyelin phosphodiesterase) as presented in Figure 2. Ceramide regeneration from complex glycosphingolipids can be regulated by either lysosomal or nonlysosomal degradation. In lysosomal degradation, catabolism of GSLs occurs by cleavage of sugar residues which leads to the formation of glucosyl ceramide and galactosylceramide. Thereafter, specific *β*-glucosidases and galactosidases hydrolyze these lipids to produce ceramide that can later be deacylated by an acid ceramidase to form sphingosine [9, 16]. The sphingosine thus produced can further be salvaged to form ceramide (the intricate details of reactions involved in the degradative pathway of complex sphingolipids are well covered in the literature and are not discussed in this review; interested readers are recommended to refer to [16] from this article and relevant citations from this reference). Defects in the function of these enzymes lead to a variety of lysosomal storage disorders such as Gaucher, Sandhoff, and Tay-Sachs diseases [20]. Degradation of sphingolipids is also an indispensable component of lipid homeostasis; consequently SM levels are maintained by the catabolic action of sphingomyelinases (also called sphingomyelin phosphodiesterases, SMPD), releasing ceramide and the corresponding head group, phosphorylcholine. 

#### 2.2.1. Compartmental View of Sphingolipid Metabolic Pathway

**Figure 3:**
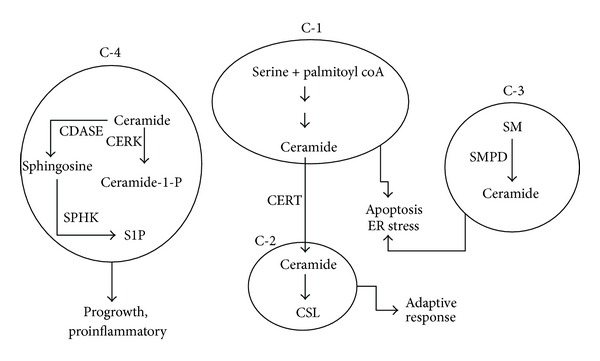
Hypothetical compartmentalization of sphingolipid metabolism. Reactions in the sphingolipid metabolic pathway can be isolated into different hypothetical compartments with each compartment representing ceramide either as a substrate or product of the reactions. Compartment C-1 is a *de novo* ceramide synthesis channel, compartment C-2 represents reactions leading to synthesis of complex sphingolipids, compartment C-3 is comprised of reaction leading to hydrolytic generation of ceramide from sphingomyelin, and compartment C-4 represents reactions in which ceramide is converted into other intermediates like sphingosine-1-phosphate and ceramide-1-phosphate. Activities/status of each compartment can be translated into different physiological events; accumulation of ceramide due to increased production of ceramide in C-1 would result in ER stress, increased generation of ceramide-1 phosphate, and sphingosine-1-phosphate in compartment C-4 results in progrowth and proinflammatory scenario in the cell. In different disease conditions, the status of each of these compartments contributes to the overall pathology of the disease.

 It is evident that sphingolipid metabolic pathway is complex and involves several reactions and cellular organelles. Outcomes of these reactions in different organelles could lead to serous physiological consequences and this forms the basis for the role of sphingolipids in disease pathogenesis. In this review we have presented sphingolipid metabolic pathway as a combination of four sets of modules as depicted in Figure 3.

- The *de novo* biosynthesis of ceramide which occurs in ER is represented by the compartment C1.
- The conversion of ceramide into complex sphingolipid like SM and glycosphingolipids is represented by compartment C2.
- Hydrolysis of SM which produces ceramide is presented as compartment C3.
- Conversion of ceramide into bioactive molecules such as C1P and S1P is represented by compartment C4.

Activities in each of these compartments can be mapped to physiological processes; for example, overall increase in the enzymes of *de novo* ceramide biosynthesis and hence increased levels of ceramide in compartment C1 can be designated as a proapoptotic scenario. Increased ceramide production in the ER followed by decreased CERT expression would result in an accumulation of ceramide in this organelle and hence can result in ER stress [21]. Decreased expression of enzymes involved in hydrolysis of complex sphingolipids as indicated in C3 can be characterized as sphingolipid storage. Increase in the components of sphingolipid rheostat (sphingosine kinase (SPHK1) and ceramide kinase (CERK)) thereby increasing the levels of C1P and S1P in compartment C4 can be taken to represent progrowth situations. Compartment C2 provides a channel wherein proapoptotic ceramide is converted into inert complex sphingolipids and this compartment can be considered as an adaptive channel since this channel is employed by drug resistant cancer cells as an adaptive mechanism. This approach provides a more explanatory vision for finding links between severe pathological diseases and sphingolipid metabolism.

## 3. Sphingolipid Metabolism in Pathogenesis of Human Diseases

Human diseases resulting due to impaired sphingolipid metabolism are generally the outcome of defect in enzymes that degrade the sphingolipids [22]. In general, they are a group of relatively rare inborn errors of metabolism resulting in accumulation of sphingolipids (sphingolipidosis) caused by defects in the genes coding for proteins taking part in the lysosomal degradation of sphingolipids [23]. Historically, the pathological significance of sphingolipid related diseases is discussed with reference to sphingolipidosis. There has been a tremendous progress in the sphingolipid research in the recent past and it is now clear that sphingolipids can play a major role in pathogenesis of several diseases apart from traditionally studied sphingolipidosis. Deregulation of sphingolipid homeostasis is established as a key factor in the pathogenesis of several disorders like metabolic, neuronal, and proliferative disorders. 

Recent studies have established the role of altered sphingolipid metabolism in brain cells in Alzheimer's disease [24]. The key role of sphingomyelinases in this disease is to promote apoptosis in neuronal cells through generation of proapoptotic molecule, ceramide [25]. In addition, serine palmitoyl transferase (SPT) has been shown to be downregulated by the amyloid precursor protein [26]. This exclusive physiological function of the amyloid precursor protein suggests the involvement of SPT and sphingolipid metabolism in Alzheimer's disease pathology.

It has been established for decades that patients with cirrhosis had increased levels of plasma long chain fatty acids (LCFA) as compared to controls, and the level of these substances is augmented with greater severity of the disease [27]. Furthermore, ceramide has been known as the prototype of sphingolipids that provokes cell death and its levels increase in response to apoptotic stimuli such as ionizing radiation or chemotherapy. In the liver, accumulation of ceramide may contribute to a variety of complications, leading to the substitution of steatosis to steatohepatitis [28], which can further develop into cirrhosis and hepatocellular carcinoma (HCC). Different studies have shown that either pharmacologic ceramide accumulation or systemic intravenous administration of liposomal ceramide is an effective approach against HCC [29].

Though a lot of research has already been done to emphasize the association of sphingolipids with several disorders, an understanding of the respective pathology in more detail with reference to sphingolipid metabolic pathway genes would be desirable. An analysis of the expression levels of genes involved in sphingolipid metabolic pathway presented below throws more light on the predisposition of sphingolipid pathway genes to execute the disease process.

Here we discuss the role of sphingolipid metabolism in three different disease conditions—type 2 diabetes mellitus (T2DM), Alzheimer's disease (AD), and hepatocellular carcinoma (HCC). For this purpose, we obtained the patterns in expression profiles of genes involved in sphingolipid metabolism under each of the diseased state from publicly available databases and mapped them to different physiological processes visualized through hypothetical compartments (Figure 3). Once the sphingolipid gene expression pattern from each study was mapped to a physiological process, possible effects of changes in the enzymes were predicted under diseased conditions. This is discussed in detail in the subsequent sections. 

### 3.1. Role of Sphingolipid Metabolism in Pathogenesis of Type 2 Diabetes Mellitus (T2DM)

T2DM is a metabolic disease characterized by insulin resistance primarily in adipose, liver, and muscle tissues. Earlier reports suggesting that sphingolipids might play a role in insulin signaling came from experiments indicating accumulation of ceramides in insulin-resistant tissues. Zucker obese rats, a common model to study insulin resistance, were found to have elevated ceramide levels within the liver and skeletal muscles [30]. Ceramide can influence insulin signaling pathway by two autonomous mechanisms: (1) through activation of protein phosphatase 2A (PP2A), which in turn can dephosphorylate and hence inhibit Akt/PKB [31] and (2) by inhibiting the translocation and activation of Akt/PKB through protein kinase C [32]. Ceramide analogs have been shown to inhibit insulin stimulated glucose uptake, GLUT4 translocation, and glycogen synthesis in cultured cells. They inhibit Akt/PKB in cultured muscle cells [33–35], adipocytes [36], and hepatocytes [37]. Decreasing the levels of ceramide by using inhibitors can restore the insulin sensitivity in lipid induced insulin resistant cell culture [33, 34]. Ceramide levels have also been reported to increase in the muscles or liver of insulin-resistant rodents [30] or human [38, 39]. S1P having progrowth properties often opposes ceramide action, allowing researchers to purport the existence of a ceramide: S1P rheostat that controls cellular responses [40]. Sphingosine kinase (SPHK) hence is an important lipid kinase that maintains the balance between progrowth and proapoptotic precursors. Recently, it has been reported that such a rheostat is very important for both islet function and beta cell survival and may act as a possible therapeutic target to protect the beta cell from diabetes related complications and to improve pancreatic islet function [41].

**Figure 4:**
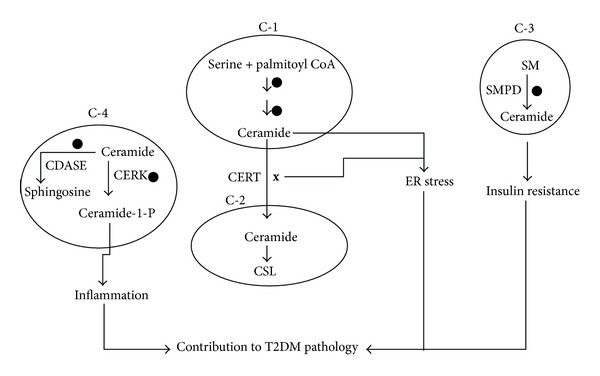
Status of different compartments in T2DM. In case of T2DM, the enzymes of the *de novo* ceramide synthesis, sphingomyelinase (SMPD), and ceramide kinase (CERK) genes are upregulated (*⚫*). CERT gene which is responsible for the transport of ceramide from ER to Golgi complex is downregulated (**x**). Consequently, this scenario might result in increased ER stress, insulin resistance, and inflammation, all of which are key pathologies associated with T2DM.

We analyzed gene expression data obtained from separate studies (GSE15653, GSE22435, and GSE22309) and Figure 4 presents the status of different hypothetical compartments C1, C2, C3, and C4 in T2DM. Genes involved in *de novo* biosynthesis of ceramide (compartment C1) show an increased level of expression in T2DM which apparently indicate that ceramide levels are increased. In general, ceramide thus formed must be transported to the Golgi apparatus with the help of CERT protein in mammals [13, 14]. However, in T2DM, genes coding for CERT protein were found to be downregulated. Overall, this might present a scenario wherein there is an increased *de novo* ceramide biosynthesis in the ER and a decreased transport of ceramide due to decreased levels of CERT. Ceramide thus accumulated in the ER is a contributing factor for ER stress. ER stress in turn is known to inhibit insulin receptor signaling through the activation of c-Jun N-terminal kinase (JNK) and subsequent serine phosphorylation of insulin receptor substrate-1 (IRS-1) [42]. Studies indicate that mice deficient in X-box-binding protein-1 (XBP-1), a transcription factor that controls the ER stress response, develop insulin resistance [43]. Apparently elevated ceramide in the ER and subsequent ER stress contributes to insulin resistance—a major pathology of T2DM.

In addition to its *de novo* route, ceramide can also be produced by hydrolysis of sphingomyelin in the membrane. This reaction is carried out by sphingomyelinase. Several cellular insults have been shown to trigger the sphingomyelin hydrolysis leading to generation of ceramide, which in turn would bring about the subsequent effects. Two proinflammatory cytokines, tumor necrosis factor alpha (TNF-*α*) and interleukin 1 beta (IL-1*β*), play an important role in hydrolytic generation and accumulation of ceramide [44, 45]. TNF-*α* has been shown to be associated with the stimulation of insulin resistance [46–48]. Neutral and acid SMPD is reported to be elevated in adipose tissue of obese rodents, possibly through a TNF-*α*-regulated mechanism [49]. In T2DM, the genes responsible for hydrolytic generation of ceramide (SMPD) are upregulated in the compartment C3. This may again result in increased ceramide levels and hence concomitantly lead to insulin resistance. 

Inflammation is one of the major components of the pathogenesis of T2DM and immunomodulatory strategies targeting inflammation are proposed as a therapeutic approach [50, 51]. Sphingolipids act as potential players in the process of inflammation and clinical data suggest a correlation between ceramide, inflammation, and insulin resistance [52, 53]. Studies have demonstrated that many of the proinflammatory effects of ceramide can be attributed to its phosphorylated derivative ceramide-1-phosphate [54]. Ceramide-1-phosphate can bind directly to phospholipase-A2 (PLA2) [55] and allosterically activate the enzyme leading to release of arachidonic acid and subsequent prostaglandin formation [56]. Ceramide kinase (CERK), a key gene involved in the generation of ceramide-1-phosphate, is upregulated in T2DM condition (Figure 4). With obesity/T2DM being a case of chronic low grade inflammation, it is possible that increased CERK activity (and consequent increase in C1P levels) would serve to execute a proinflammatory scenario contributing towards pathology. Recently, the CERK null mice have been shown to be resistant to diet-induced obesity and glycemic dysregulation [57].

Overall in the context of T2DM, sphingolipid metabolic pathway contributes to ER stress and inflammation, two major contributors for insulin resistance.

### 3.2. Role of Sphingolipid Metabolism in Pathogenesis of Alzheimer's Disease

 Sphingolipids form the integral component of the brain and a proper sphingolipid homeostasis is essential for the normal functioning of neurons. Several neurological disorders like Niemann-Pick disease (type I), Gaucher's disease, and Tay-Sacks disease result due to impaired activities of the enzymes that handle complex sphingolipids [14]. Studies suggest that even minor changes in sphingolipid balance may play significant roles in the development of neurodegenerative diseases including Alzheimer's disease [58], amyotrophic lateral sclerosis [59], Parkinson's disease [60], and dementia [61].

Alzheimer's disease (AD) is a neurodegenerative disorder characterized by a progressive decline in cognitive processes gradually leading to dementia. Extracellular deposition of A*β* peptide and neurofibrillary tangles are the well-known histopathological markers of AD [62]. Accumulation of abnormally folded A*β* in association with inability to catabolize A*β* peptide triggers the neuronal degeneration and this event is critical to the development of AD [63]. Sphingolipids are known to contribute to the development of AD at various steps during the progression of the disease. 

**Figure 5:**
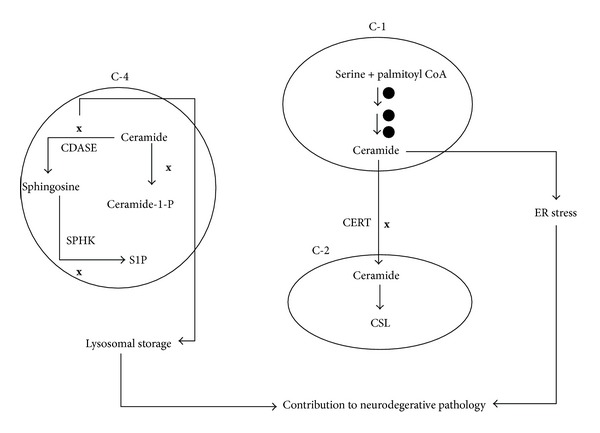
Status of different compartments in Alzheimer's disease. In Alzheimer's disease condition, there is an upregulation of genes involved in the *de novo* ceramide synthesis (*⚫*) and downregulation of CERT (**x**). This contributes towards ER stress. CERK gene which generates C1P a progrowth molecule is downregulated (**x**). Down regulation of ceramidase might lead to accumulation of ceramide which is a proapoptotic molecule. Overall this scenario would result in ER stress and proapoptotic phenotype.

We analyzed data obtained from gene expression studies (GSE29652, GSE5281, GSE16759, GDS1979, GSE34879, GSE30945, GSE15222, GSE4757, and GSE28146) and as depicted in Figure 5, *de novo* synthesis of ceramide (indicated by compartment C1) is increased significantly and CERT gene which is involved in transport of ceramide from ER to Golgi complex is downregulated. The overall effect may lead to ER stress due to ceramide accumulation in the ER. ER is an organelle involved in proper folding and sorting of proteins. Impaired protein folding and subsequent accumulation of neurotoxic peptides are major pathological factors in AD. Neuronal cells are vulnerable to perturbations which affect the homeostasis of ER and disturbances in redox and Ca2+ balances [64]. A number of studies have demonstrated ER stress as a major pathological driver in several neurodegenerative diseases [65–68]. Immunohistochemical studies have indicated that neurons of AD patients show prominent expression of ER stress markers [66]. The presence of oxidative stress, accumulation of neurofibrillary tangles, and intraneuronal amyloid-*β* aggregates [69] in AD point out the role of ER stress in this disease [70, 71]. It has been established that higher concentration of ceramide promotes the A*β* biogenesis by stabilizing the APP cleaving enzyme [72, 73], but diminished concentration of ceramide leads to a reduction in the secretion of APP and A*β* in human neuroblastoma cells [74]. Thus, it is imperative that ceramide and A*β* may work together to encourage neuronal death in AD. 

CERK and ceramidase (CDASE) are also downregulated in AD (compartment C4), which indicate decreased levels of progrowth molecule ceramide 1 phosphate and accumulation of ceramide. Diminished levels of C1P hence might reinforce a proapoptotic/degenerative scenario—a hall mark of AD. The decreased activity of ceramidase can reflect a scenario wherein ceramide accumulation in the lysosomes could lead to lysosomal dysfunction. In fact, lysosomal storage defect (LSD) is considered as one of the early histological changes associated with AD [75]. Accumulation of sphingolipids not only underlies the pathogenesis of LSDs but also elicits increased generation of A*β* and contributes to neurodegeneration in AD. Experimental evidences indicate that accumulation of sphingolipids decreases the lysosome dependent degradation of APP-CTFs (amyloid precursor protein c-terminal fragments) and increases *γ*-secretase activity. Both these activities result in increased generation of intracellular and secreted A*β* [74]. 

Thus, from sphingolipid perceptive, AD may be envisaged as an ER stress and lysosomal storage disorder arising through increased ceramide synthesis and decreased catabolism. Increased ceramide production due to increased *de novo* biosynthesis of ceramide can be a contributing factor for neurodegeneration. An earlier study reports that genes controlling *de novo *synthesis of ceramide are upregulated at early stages in AD disease progression [76]. Sphingosine, a proapoptotic sphingolipid is known to be accumulated in AD brain [77, 78]. One study has specified decreased S1P levels in cytosolic fractions of grey matter from the frontotemporal areas of AD patients [78]. 

## 4. Role of Sphingolipid Metabolism in Pathogenesis of Hepatocellular Carcinoma

Impaired sphingolipid homeostasis is a common theme for most of the cancers [79] and overexpression of SPHK1 has been identified in multiple cancer cells derived from breast, colon, lung, ovary, stomach, uterus, kidney, and rectum [80]. Central to the role of sphingolipids in cancer is the fact that ceramide generated through either the *de novo*/hydrolytic pathway is a proapoptotic molecule and sphingosine-1-phosphate and ceramide 1 phosphate resist the action of ceramide and promote cell proliferation [81]. Owing to the significant role of sphingolipids in cell death/survival regulation, the alteration in sphingolipid metabolism has a profound impact on cancer biology and therapy. While increased levels of progrowth sphingolipids would enhance the cell proliferation, channelizing the proapoptotic ceramide into other sphingolipid molecules would augment resistance to drug induced cell death. In fact many cancer cells suppress their ceramide biosynthetic machinery while upregulating the biosynthesis of progrowth sphingolipids like S1P. This modulation would serve to increase proliferation of these cells. Besides maintaining a proliferative phenotype, many cancer cell types resist therapy in several ways including escape from therapy-induced apoptosis [82]. Decreasing the levels of proapoptotic ceramide in the cells is one of the adaptive means to resist therapy-induced apoptosis. 

**Figure 6:**
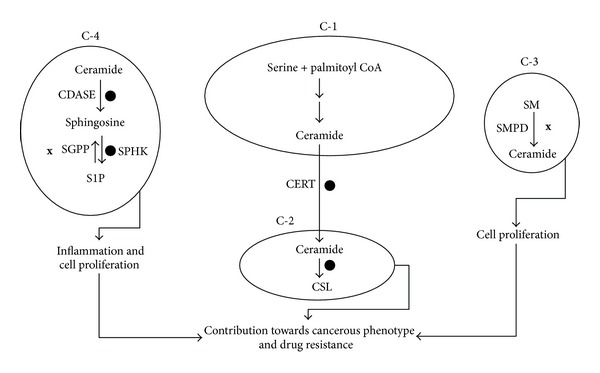
Status of different compartments in hepatocellular carcinoma. Hepatocellular carcinoma is a case of proliferative scenario mediated through downregulation of ceramide production (in compartment C-3(**x**)) and upregulation (*⚫*) of complex sphingolipid biosynthesis in order to quench the proapoptotic ceramide.

Hepatocellular carcinoma is a common type of liver cancer resulting due to either viral causes or cirrhosis. We analyzed gene expression data obtained from several studies (GSE21362, GSE6764, GSE14323, GSE5975, GSE25097, GSE10459, and GSE14323) and Figure 6 demonstrates the status of four compartments in hepatocellular carcinoma (HCC). The expression of genes involved in the *de novo* ceramide synthesis is unaltered as depicted by compartment C1 and levels of CERT are upregulated. In compartment C2, markedly, there is an upregulation of the enzymes involved in the synthesis of complex sphingolipids. In compartment C3, hydrolytic generation of ceramide from sphingomyelin is downregulated once again ensuring lesser levels of ceramide. 

The digression of proapoptotic ceramide to progrowth molecule S1P may modulate the future of a cell in response to cancer therapy [83, 84]. Ceramidases promote carcinogenesis by disturbing the ceramide/S1P ratio, permitting phosphorylation of sphingosine by SPHKs, and determine the efficacy of cancer therapy. For instance, inhibition of an acid CDASE by a newly developed ceramide analogue, B13, induces apoptosis in cultured human colon cancer cells and prevents liver metastases *in vivo* [85]. Concurrent to these findings, our analysis also revealed that, in compartment C4, the expression of CDASEs and SPHK, enzymes involved in the conversion of ceramide to sphingosine and later to sphingosine-1-phosphate are upregulated. Sphingosine-1-phosphate phosphatase (SGPP), an enzyme involved in dephosphorylation of progrowth molecule sphingosine-1-phosphate to proapoptotic sphingosine, was found to be downregulated. Apparently in HCC, sphingolipid metabolic pathway is driven towards decreasing the levels of ceramide and increasing progrowth sphingolipids. Increased expression of enzymes involved in the complex sphingolipid biosynthesis in compartment C2 serves as an adaptive channel to quench the proapoptotic ceramide. Elevated expression of CERT apparently will augment the transfer of ceramide from ER to Golgi complex, providing it as a substrate for complex sphingolipid biosynthesis. Earlier studies employing different cancer cell types have established the relationship between glucosyl ceramide synthase and chemoresistance. In studies comparing the nature of lipid intermediates between drug-sensitive and resistant cancer cell lines, glucosyl ceramide levels were found to be higher in drug-resistant cells [86, 87]. The role of GCS (glucosyl ceramide synthase) is further confirmed in cell model where overexpression of GCS resulted in resistance of HL-60 to doxorubicin-induced apoptosis [88].

In summary, in HCC sphingolipid, metabolism is manipulated at 3 levels.

1. Channels generating the proapoptotic ceramide through hydrolytic machinery are decreased.
2. The channel converting the proapoptotic ceramide into progrowth molecule is upregulated.
3. Adaptive channel which converts proapoptotic ceramide into less effective complex sphingolipids is activated.

While the first two changes ensure that cell assumes a proliferative mode favoring the cancer phenotype, the adaptive channel in which proapoptotic ceramide is quenched into less effective complex sphingolipids contributes towards drug resistance. 

## 5. Conclusions

Sphingolipids affect various aspects of cell physiology like cell proliferation, cell death, differentiation, and cell signaling and are known to contribute to key cellular pathologies like ER stress, insulin resistance, inflammation, and drug resistance. Analyzing the gene expression pattern in three different disease conditions—T2DM, Alzheimer's disease, and hepatocellular carcinoma, indicates that under different disease conditions sphingolipid metabolic pathway may employ slightly different routes to contribute towards the pathology. For example, in diabetes, sphingolipid metabolic pathway genes are manipulated in such a way that it leads to ER stress and inflammation, while Alzheimer's disease condition is a case of increased ceramide mediated apoptosis coupled with inability to degrade ceramide. In hepatocellular carcinoma, sphingolipid pathway is manipulated in such a way that reactions leading to quenching of proapoptotic molecule ceramide into inert complex sphingolipids and those leading to generation of progrowth sphingolipids are upregulated. Although a bias for the sphingolipid metabolic pathway is apparent under these disease conditions, further analysis is required to verify if such a phenomenon is universally applicable. Understanding the nature of changes in the sphingolipid metabolic pathway and their overall pathological effect under different disease conditions would be very useful in order to design therapeutic strategies. 

## 6. Highlights

- The sphingolipid metabolic pathway is a major player in the pathology of human diseases.
- This review focuses on changes in the sphingolipid pathway in 3 different diseases.
- In metabolic disorder, this pathway contributes to endoplasmic reticulum stress (ER stress) and inflammation.
- In Alzheimer's disease, the pathway contributes to ER stress and apoptosis.
- In hepatocellular carcinoma, the pathway contributes to proproliferative scenario.

## Abbreviation

AD: Alzheimer's disease
APP: Amyloid precursor protein
A*β*: Amyloid beta
C1P: Ceramide-1-phosphate
CDASE: Ceramidase
CERK: Ceramide kinase
CERT: Ceramide transfer protein
COX-2: Cyclooxygenase-2
ER: Endoplasmic reticulum
GLUT-4: Glucose transporter-4
GSL: Glycosphingolipid
HCC: Hepatocellular carcinoma
IL-1*β*: Interleukin-1*β*
IRS-1: Insulin receptor substrate-1
JNK: c-Jun N-terminal kinase
LCFA: Long chain fatty acids
LSD: Lysosomal storage disorder
PGE-2: Prostaglandin E-2
PKB: Protein kinase B
PLA-2: Phospholipase A-2
PP2A: Protein phosphatase 2A
S1P: Sphingosine-1-phosphate
SL: Sphingolipid
SM: Sphingomyelin
SMPD: Sphingomyelin phosphodiesterase
SPHK: Sphingosine kinase
SPT: Serine palmitoyltransferase
T2DM: Type 2 diabetes mellitus
XBP-1: X-box binding protein-1.

## References

[1] Chen Y, Liu Y, Sullards MC, Merrill AH (2010). An introduction to sphingolipid metabolism and analysis by new technologies. *NeuroMolecular Medicine*.

[2] Merrill AH, Wang MD, Park M, Sullards MC (2007). (Glyco)sphingolipidology: an amazing challenge and opportunity for systems biology. *Trends in Biochemical Sciences*.

[3] Hannun YA, Obeid LM (2002). The ceramide-centric universe of lipid-mediated cell regulation: stress encounters of the lipid kind. *Journal of Biological Chemistry*.

[4] Ohanian J, Ohanian V (2001). Sphingolipids in mammalian cell signalling. *Cellular and Molecular Life Sciences*.

[5] Holthuis JCM, Pomorski T, Raggers RJ, Sprong H, van Meer G (2001). The organizing potential of sphingolipids in intracellular membrane transport. *Physiological Reviews*.

[6] Brice SE, Cowart LA (2011). Sphingolipid metabolism and analysis in metabolic disease. *Advances in Experimental Medicine and Biology*.

[7] Alvarez-Vasquez F, Sims KJ, Cowart LA, Okamoto Y, Voit EO, Hannun YA (2005). Simulation and validation of modelled sphingolipid metabolism in *Saccharomyces cerevisiae*. *Nature*.

[8] Hannun YA, Luberto C (2004). Lipid metabolism: ceramide transfer protein adds a new dimension. *Current Biology*.

[9] Hannun YA, Obeid LM (2008). Principles of bioactive lipid signalling: lessons from sphingolipids. *Nature Reviews Molecular Cell Biology*.

[10] Hanada K (2003). Serine palmitoyltransferase, a key enzyme of sphingolipid metabolism. *Biochimica et Biophysica Acta*.

[11] Acharya U, Acharya JK (2005). Enzymes of sphingolipid metabolism in *Drosophila melanogaster*. *Cellular and Molecular Life Sciences*.

[12] Sugimoto Y, Sakoh H, Yamada K (2004). IPC synthase as a useful target for antifungal drugs. *Current Drug Targets*.

[13] Hanada K, Kumagai K, Tomishige N, Yamaji T (2009). CERT-mediated trafficking of ceramide. *Biochimica et Biophysica Acta*.

[14] Rao RP, Acharya JK (2008). Sphingolipids and membrane biology as determined from genetic models. *Prostaglandins and Other Lipid Mediators*.

[15] Zeidan YH, Hannun YA (2007). Translational aspects of sphingolipid metabolism. *Trends in Molecular Medicine*.

[16] Kolter T, Sandhoff K (2005). Principles of lysosomal membrane digestion: stimulation of sphingolipid degradation by sphingolipid activator proteins and anionic lysosomal lipids. *Annual Review of Cell and Developmental Biology*.

[17] Snook CF, Jones JA, Hannun YA (2006). Sphingolipid-binding proteins. *Biochimica et Biophysica Acta*.

[18] Summers SA (2006). Ceramides in insulin resistance and lipotoxicity. *Progress in Lipid Research*.

[19] Menaldino DS, Bushnev A, Sun A (2003). Sphingoid bases and de novo ceramide synthesis: enzymes involved, pharmacology and mechanisms of action. *Pharmacological Research*.

[20] Schulze H, Sandhoff K (2011). Lysosomal lipid storage diseases. *Cold Spring Harbor Perspectives in Biology*.

[21] Wang X, Rao RP, Kosakowska-Cholody T (2009). Mitochondrial degeneration and not apoptosis is the primary cause of embryonic lethality in ceramide transfer protein mutant mice. *Journal of Cell Biology*.

[22] Kolter T, Sandhoff K (2006). Sphingolipid metabolism diseases. *Biochimica et Biophysica Acta*.

[23] Raas-Rothschild A, Pankova-Kholmyansky I, Kacher Y, Futerman AH (2004). Glycosphingolipidoses: beyond the enzymatic defect. *Glycoconjugate Journal*.

[24] Haughey NJ, Bandaru VVR, Bae M, Mattson MP (2010). Roles for dysfunctional sphingolipid metabolism in Alzheimer’s disease neuropathogenesis. *Biochimica et Biophysica Acta*.

[25] Jana A, Pahan K (2010). Fibrillar amyloid-*β*-activated human astroglia kill primary human neurons via neutral sphingomyelinase: implications for Alzheimer’s disease. *Journal of Neuroscience*.

[26] Grimm MOW, Grösgen S, Rothhaar TL (2011). Intracellular APP domain regulates serine-palmitoyl-CoA transferase expression and is affected in Alzheimer’s disease. *International Journal of Alzheimer’s Disease*.

[27] Wilcox HG, Dunn GD, Schenker S (1978). Plasma long chain fatty acids and esterified lipids in cirrhosis and hepatic encephalopathy. *American Journal of the Medical Sciences*.

[28] Longato L, Ripp K, Setshedi M (2012). Insulin resistance, ceramide accumulation, and endoplasmic reticulum stress in human chronic alcohol-related liver disease. *Oxidative Medicine and Cellular Longevity*.

[29] Morales A, Marí M, García-Ruiz C, Colell A, Fernández-Checa JC (2012). Hepatocarcinogenesis and ceramide/cholesterol metabolism. *Anti-Cancer Agents in Medicinal Chemistry*.

[30] Turinsky J, O’Sullivan DM, Bayly BP (1990). 1,2-diacylglycerol and ceramide levels in insulin-resistant tissues of the rat in vivo. *Journal of Biological Chemistry*.

[31] Resjö S, Göransson O, Härndahl L, Zolnierowicz S, Manganiello V, Degerman E (2002). Protein phosphatase 2A is the main phosphatase involved in the regulation of protein kinase B in rat adipocytes. *Cellular Signalling*.

[32] Powell DJ, Hajduch E, Kular G, Hundal HS (2003). Ceramide disables 3-phosphoinositide binding to the pleckstrin homology domain of protein kinase B (PKB)/Akt by a PKC*ζ*-dependent mechanism. *Molecular and Cellular Biology*.

[33] Chavez JA, Knotts TA, Wang L-P (2003). A role for ceramide, but not diacylglycerol, in the antagonism of insulin signal transduction by saturated fatty acids. *Journal of Biological Chemistry*.

[34] Powell DJ, Turban S, Gray A, Hajduch E, Hundal HS (2004). Intracellular ceramide synthesis and protein kinase C*ζ* activation play an essential role in palmitate-induced insulin resistance in rat L6 skeletal muscle cells. *Biochemical Journal*.

[35] Schmitz-Peiffer C, Craig DL, Biden TJ (1999). Ceramide generation is sufficient to account for the inhibition of the insulin-stimulated PKB pathway in C2C12 skeletal muscle cells pretreated with palmitate. *Journal of Biological Chemistry*.

[36] Summers SA, Garza LA, Zhou H, Birnbaum MJ (1998). Regulation of insulin-stimulated glucose transporter GLUT4 translocation and Akt kinase activity by ceramide. *Molecular and Cellular Biology*.

[37] Holland WL, Knotts TA, Chavez JA, Wang L-P, Hoehn KL, Summers SA (2007). Lipid mediators of insulin resistance. *Nutrition Reviews*.

[38] Adams JM, Pratipanawatr T, Berria R (2004). Ceramide content is increased in skeletal muscle from obese insulin-resistant humans. *Diabetes*.

[39] Straczkowski M, Kowalska I, Nikolajuk A (2004). Relationship between insulin sensitivity and sphingomyelin signaling pathway in human skeletal muscle. *Diabetes*.

[40] Bikman BT, Summers SA (2011). Ceramides as modulators of cellular and whole-body metabolism. *Journal of Clinical Investigation*.

[41] Jessup CF, Bonder CS, Pitson SM, Coates PTH (2011). The sphingolipid rheostat: a potential target for improving pancreatic islet survival and function. *Endocrine, Metabolic and Immune Disorders*.

[42] Lee YH, Giraud J, Davis RJ, White MF (2003). c-Jun N-terminal kinase (JNK) mediates feedback inhibition of the insulin signaling cascade. *Journal of Biological Chemistry*.

[43] Özcan U, Cao Q, Yilmaz E (2004). Endoplasmic reticulum stress links obesity, insulin action, and type 2 diabetes. *Science*.

[44] Dressler KA, Mathias S, Kolesnick RN (1992). Tumor necrosis factor-*α* activates the sphingomyelin signal transduction pathway in a cell-free system. *Science*.

[45] Wiegmann K, Schütze S, Machleidt T, Witte D, Krönke M (1994). Functional dichotomy of neutral and acidic sphingomyelinases in tumor necrosis factor signaling. *Cell*.

[46] Hotamisligil GS, Shargill NS, Spiegelman BM (1993). Adipose expression of tumor necrosis factor-*α*: direct role in obesity-linked insulin resistance. *Science*.

[47] Hofmann C, Lorenz K, Braithwaite SS (1994). Altered gene expression for tumor necrosis factor-*α* and its receptors during drug and dietary modulation of insulin resistance. *Endocrinology*.

[48] Stephens JM, Lee J, Pilch PF (1997). Tumor necrosis factor-*α*-induced insulin resistance in 3T3-L1 adipocytes is accompanied by a loss of insulin receptor substrate-1 and GLUT4 expression without a loss of insulin receptor-mediated signal transduction. *Journal of Biological Chemistry*.

[49] Samad F, Hester KD, Yang G, Hannun YA, Bielawski J (2006). Altered adipose and plasma sphingolipid metabolism in obesity: a potential mechanism for cardiovascular and metabolic risk. *Diabetes*.

[50] Donath MY, Shoelson SE (2011). Type 2 diabetes as an inflammatory disease. *Nature Reviews Immunology*.

[51] Shoelson SE, Lee J, Goldfine AB (2006). Inflammation and insulin resistance. *Journal of Clinical Investigation*.

[52] de Mello VDF, Lankinen M, Schwab U (2009). Link between plasma ceramides, inflammation and insulin resistance: association with serum IL-6 concentration in patients with coronary heart disease. *Diabetologia*.

[53] Gill JMR, Sattar N (2009). Ceramides: a new player in the inflammation-insulin resistance paradigm?. *Diabetologia*.

[54] Gómez-Munoz A, Gangoiti P, Granado MH, Arana L, Ouro A (2010). Ceramide-1-phosphate in cell survival and inflammatory signaling. *Advances in Experimental Medicine and Biology*.

[55] Subramanian P, Vora M, Gentile LB, Stahelin RV, Chalfant CE (2007). Anionic lipids activate group IVA cytosolic phospholipase A2 via distinct and separate mechanisms. *Journal of Lipid Research*.

[56] Pettus BJ, Bielawska A, Spiegel S, Roddy P, Hannun YA, Chalfant CE (2003). Ceramide kinase mediates cytokine- and calcium ionophore-induced arachidonic acid release. *Journal of Biological Chemistry*.

[57] Mitsutake S, Date T, Yokota H, Sugiura M, Kohama T, Igarashi Y (2012). Ceramide kinase deficiency improves diet-induced obesity and insulin resistance. *FEBS Letters*.

[58] Mielke MM, Lyketsos CG (2010). Alterations of the sphingolipid pathway in Alzheimer’s disease: new biomarkers and treatment targets?. *NeuroMolecular Medicine*.

[59] Cutler RG, Pedersen WA, Camandola S, Rothstein JD, Mattson MP (2002). Evidence that accumulation of ceramides and cholesterol esters mediates oxidative stress-induced death of motor neurons in amyotrophic lateral sclerosis. *Annals of Neurology*.

[60] France-Lanord V, Brugg B, Michel PP, Agid Y, Ruberg M (1997). Mitochondrial free radical signal in ceramide-dependent apoptosis: a putative mechanism for neuronal death in Parkinson’s disease. *Journal of Neurochemistry*.

[61] Haughey NJ, Cutler RG, Tamara A (2004). Perturbation of sphingolipid metabolism and ceramide production in HIV-dementia. *Annals of Neurology*.

[62] Tamboli IY, Hampel H, Tien NT (2011). Sphingolipid storage affects autophagic metabolism of the amyloid precursor protein and promotes A*β* generation. *Journal of Neuroscience*.

[63] Murphy MP, Levine H (2010). Alzheimer’s disease and the amyloid-*β* peptide. *Journal of Alzheimer’s Disease*.

[64] Salminen A, Kauppinen A, Suuronen T, Kaarniranta K, Ojala J (2009). ER stress in Alzheimer’s disease: a novel neuronal trigger for inflammation and Alzheimer’s pathology. *Journal of Neuroinflammation*.

[65] Lindholm D, Wootz H, Korhonen L (2006). ER stress and neurodegenerative diseases. *Cell Death and Differentiation*.

[66] Hoozemans JJM, van Haastert ES, Nijholt DAT, Rozemuller AJM, Eikelenboom P, Scheper W (2009). The unfolded protein response is activated in pretangle neurons in Alzheimer’s disease hippocampus. *American Journal of Pathology*.

[67] Katayama T, Imaizumi K, Manabe T, Hitomi J, Kudo T, Tohyama M (2004). Induction of neuronal death by ER stress in Alzheimer’s disease. *Journal of Chemical Neuroanatomy*.

[68] Resende R, Ferreiro E, Pereira C, Oliveira CR (2008). ER stress is involved in A*β*-induced GSK-3*β* activation and tau phosphorylation. *Journal of Neuroscience Research*.

[69] Selkoe DJ (2001). Alzheimer’s disease: genes, proteins, and therapy. *Physiological Reviews*.

[70] Tanzi RE, Bertram L (2005). Twenty years of the Alzheimer’s disease amyloid hypothesis: a genetic perspective. *Cell*.

[71] LaFerla FM, Green KN, Oddo S (2007). Intracellular amyloid-*β* in Alzheimer’s disease. *Nature Reviews Neuroscience*.

[72] Patil S, Melrose J, Chan C (2007). Involvement of astroglial ceramide in palmitic acid-induced Alzheimer-like changes in primary neurons. *European Journal of Neuroscience*.

[73] Puglielli L, Ellis BC, Saunders AJ, Kovacs DM (2003). Ceramide stabilizes *β*-site amyloid precursor protein-cleaving enzyme 1 and promotes amyloid *β*-peptide biogenesis. *Journal of Biological Chemistry*.

[74] Tamboli IY, Prager K, Barth E, Heneka M, Sandhoff K, Walter J (2005). Inhibition of glycosphingolipid biosynthesis reduces secretion of the *β*-amyloid precursor protein and amyloid *β*-peptide. *Journal of Biological Chemistry*.

[75] Nixon RA, Cataldo AM, Mathews PM (2000). The endosomal-lysosomal system of neurons in Alzheimer’s disease pathogenesis: a review. *Neurochemical Research*.

[76] Katsel P, Li C, Haroutunian V (2007). Gene expression alterations in the sphingolipid metabolism pathways during progression of dementia and Alzheimer’s disease: a shift toward ceramide accumulation at the earliest recognizable stages of Alzheimer’s disease?. *Neurochemical Research*.

[77] Huang Y, Tanimukai H, Liu F, Iqbal K, Grundke-Iqbal I, Gong C-X (2004). Elevation of the level and activity of acid ceramidase in Alzheimer’s disease brain. *European Journal of Neuroscience*.

[78] He X, Huang Y, Li B, Gong C-X, Schuchman EH (2010). Deregulation of sphingolipid metabolism in Alzheimer’s disease. *Neurobiology of Aging*.

[79] Ryland LK, Fox TE, Liu X, Loughran TP, Kester M (2011). Dysregulation of sphingolipid metabolism in cancer. *Cancer Biology and Therapy*.

[80] Huang WC, Chen CL, Lin YS, Lin CF (2011). Apoptotic sphingolipid ceramide in cancer therapy. *Journal of Lipids*.

[81] Oskouian B, Saba JD (2010). Cancer treatment strategies targeting sphingolipid metabolism. *Advances in Experimental Medicine and Biology*.

[82] Gottesman MM (2002). Mechanisms of cancer drug resistance. *Annual Review of Medicine*.

[83] Kolesnick R (2002). The therapeutic potential of modulating the ceramide/sphingomyelin pathway. *Journal of Clinical Investigation*.

[84] Ogretmen B, Hannun YA (2004). Biologically active sphingolipids in cancer pathogenesis and treatment. *Nature Reviews Cancer*.

[85] Selzner M, Bielawska A, Morse MA (2001). Induction of apoptotic cell death and prevention of tumor growth by ceramide analogues in metastatic human colon cancer. *Cancer Research*.

[86] Lavie Y, Cao H-T, Bursten SL, Giuliano AE, Cabot MC (1996). Accumulation of glucosylceramides in multidrug-resistant cancer cells. *Journal of Biological Chemistry*.

[87] Morjani H, Aouali N, Belhoussine R, Veldman RJ, Levade T, Manfait M (2001). Elevation of glucosylceramide in multidrug-resistant cancer cells and accumulation in cytoplasmic droplets. *International Journal of Cancer*.

[88] Itoh M, Kitano T, Watanabe M (2003). Possible role of ceramide as an indicator of chemoresistance: decrease of the ceramide content via activation of glucosylceramide synthase and sphingomyelin synthase in chemoresistant leukemia. *Clinical Cancer Research*.

